# Efficacy of autoantibodies combined with tumor markers in the detection of lung cancer

**DOI:** 10.1002/jcla.24504

**Published:** 2022-05-21

**Authors:** Yinyu Mu, Jing Li, Fuyi Xie, Lin Xu, Guodong Xu

**Affiliations:** ^1^ Department of Laboratory Medicine, Ningbo Medical Center, Lihuili Hospital Ningbo University Ningbo China; ^2^ Department of Cardiothoracic Surgery, Ningbo Medical Center, Lihuili Hospital Ningbo University Ningbo China

**Keywords:** autoantibody, combination, diagnosis, lung cancer, tumor markers

## Abstract

**Background:**

The purpose of this study was to explore the detection value of seven autoantibodies (TAAbs): p53, PGP9.5, SOX2, GBU4‐5, MAGE A1, CAGE, and GAGE7 and three tumor markers: CYFRA21‐1, NSE, and SCCA in the diagnosis of lung cancer.

**Methods:**

ELISA was used to detect the levels of the TAAbs, and chemiluminescence immunoassay was used to test the levels of the tumor markers. The diagnostic efficacy of the TAAbs combined with the tumor markers for lung cancer was evaluated by receiver operating characteristic (ROC) curves.

**Results:**

The positive rate of the combined detection of seven TAAbs and three tumor markers in lung cancer (37.8%) was higher than that in other three groups. The positive rates of SOX2, GAGE7, MAGE A1, CAGE, CYFRA21‐1, and SCCA had differences among the four groups. Compared with the benign lung disease group, only GAGE7, CYFRA21‐1, and SCCA differed among the groups. The combined sensitivity of the TAAbs was 29.07% (AUC, 0.594), the combined sensitivity of all the markers was 37.76% (AUC, 0.660 [*p* < 0.05]), and Youden's index was 0.196. In the lung cancer group, CYFRA21‐1 had a significant difference in age and sex, and SOX2, MAGE A1, CYFRA21‐1, NSE, and SCCA were significantly different in pathological type and TNM. In contrast, p53 and GBU4‐5 showed no significant differences in age, sex, pathological type, and TNM.

**Conclusions:**

The combined detection of seven TAAbs and three tumor markers could be useful in early diagnosis of lung cancer.

## Graphical abstract

Seven autoantibodies combined with three tumor markers was more effective in diagnosing LC than either alone or in combination with seven autoantibodies. GAGE7, CYFRA21‐1 and SCCA were different between the LC and the lung disease group, and the results of the combination of seven autoantibodies or the combination of seven autoantibodies and three tumor markers were statistically significant between four groups or LC and lung disease group.

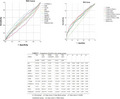

## 1 INTRODUCTION

Lung cancer (LC) is one of the most common cancers in the world and is the primary cause of cancer‐related death.^1^ According to the pathological type, LC can be categorized into small cell LC (SCLC) and non‐small cell LC (NSCLC). NSCLC includes squamous cell carcinoma (LSC), adenocarcinoma (LAC), and large cell cancer, among which LAC accounts for approximately 80%.^2^, ^3^ Although the current mortality rate of LC has decreased due to the popularization of health examination and the advancement of diagnostic technology, it remains at a relatively high level, and the 5‐year survival rate is unsatisfactory.^4^ The occurrence of cancer is related to many factors, such as environmental pollution, gene mutation, heredity, and so on. Gene mutation and recombination occur in cells in the early stage of disease, and related antigens are released. These antigens are recognized by the immune system, which produces antibodies against the antigen as autoantibodies for lung cancer. The autoantibodies can be detected in the blood during the first 5 years of imaging diagnosis of LC due to the sensitivity and stability of the immune system.^5^ Tumor markers are specific substances produced and released by tumor cells, which often exist in tumor cells or host body fluids in the form of metabolites, such as antigens, enzymes, and hormones. For example, CYFRA21‐1, NSE, and SCCA are used as LC markers and play an important role in the diagnosis of LC. At present, the commonly used diagnostic techniques for LC include imaging examinations, CT‐guided lung biopsy, molecular biology techniques, and serological examinations of autoantibodies and tumor markers. However, imaging examinations result in difficulty in determining the nature of nodules at an early stage and radiation. Additionally, CT‐guided lung biopsy is an invasive operation with risks. In addition, the disadvantage of low dose CT scan is too many false‐positive results making subsequent medical procedures costlier. Moreover, molecular biology techniques, such as gene sequencing, possess a low application rate due to their high cost.^6^ In contrast, the advantages of serum autoantibodies and tumor markers in the diagnosis of LC have recently become clear.

There are many TAAbs of LC, including p53, PGP9.5, SOX2, GBU4‐5, MAGE A1, CAGE, and GAGE7. Specifically, p53 is a tumor suppressor gene involved in regulating the cell cycle.^7^, ^8^ PGP9.5 is a ubiquitinase expressed in neural tissue and various malignant tumors, including LC cells.^9^, ^10^ MAGE A1 belongs to the human melanoma antigen family and is a special tumor antigen that is thought to be involved in the occurrence of various tumors. In recent years, studies have shown that MAGE A1 was closely related to LC prognosis. Yi et al. collected bone marrow of patients with LC and used real‐time quantitative reverse transcription‐polymerase chain reaction (RT‐PCR) technology to find that the expression of MAGE A1‐6 was closely related to poor prognosis. Therefore, they identified MAGE A1‐6 as a new prognostic factor for clinical reference.^11^ Furthermore, research by Mao et al.^12^ showed that MAGE A1 had malignant behavior in LC, which may provide a new target for LC treatment. SOX2 is a transcription factor, belonging to the SOX family. SOX was found to be involved in the proliferation and development of various cancers, and its expression was upregulated in several cancers. Hence, SOX may also be used as an index to evaluate prognosis.^13^ Finally, neuron‐specific enolase (NSE) was found to be highly expressed in LC, especially SCLC.^14^ In recent years, many studies have shown that NSE is related to the diagnosis and staging of LC.^15^

However, single tumor marker detection possesses lower sensitivity and specificity in the early diagnosis of LC, which has little application value in the early screening and diagnosis of LC, and the combined detection of multiple markers can make up for these defects.^16^, ^17^ So, it is important to find a better screening method to improve diagnostic rate of LC. In recent years, we have found that the positive rate of seven TAAbs in patients with early LC was significantly higher than those in patients with benign lung disease and healthy controls, but the sensitivity was low.^18^ Hence, we speculated that the combination of these seven TAAbs and tumor markers might improve the accuracy of diagnosis of LC. The aim of this study was to evaluate the clinical value of these seven TAAbs and tumor markers in the screening and diagnosis of LC.

## 2 MATERIALS AND METHODS

### 2.1 Sample information

We collected 780 patients with pulmonary nodules at Lihuili Hospital of Ningbo Medical Center from January 2018 to December 2021, including 633 patients diagnosed with LC and 147 patients with benign lung disease. During the same period, we also collected 211 healthy people and 89 patients with tumors in other sites. Among 633 patients with LC, 314 were male with an average age of 60 years (range, 17–83 years), and 319 were female with an average age of 59 years (range, 25–85 years). Among the 147 patients with benign lung disease, 84 were male with an average age of 59 years (range, 23–85 years), and 63 were female with an average age of 57 years (range, 27–78 years). Among the 211 healthy people, 135 were male with an average age of 50 years (range, 26–87 years), and 76 were female with an average age of 47 years (range, 28–76 years). Finally, of the 89 patients with tumors in other sites, 65 were male with an average age of 63 years (range, 25–81 years), and 24 were female with an average age of 63 years (range, 38–89 years) (Table 1). The benign lung disease and healthy examination groups were referred to as the control group (CG). This study was reviewed and approved by the ethics committee of Ningbo Lihuili Hospital.

**TABLE 1:** Characteristics of study population

Features	Total (*n* = 1080)	Lung cancer (*n* = 633)	Healthy examination (*n* = 211)	Benign lung disease (*n* = 147)	Other tumor group (*n* = 89)	*p*
Age (years)						
≥60	548 (50.7%)	375 (59.2%)	37 (17.5%)	74 (50.3%)	62 (69.7%)	0.000
<60	532 (49.3%)	258 (40.8%)	174 (82.5%)	73 (49.7%)	27 (30.3%)	
Sex						
Male	598 (55.4%)	314 (49.6%)	135 (64.0%)	84 (57.1%)	65 (73.0%)	0.000
Female	482 (44.6)%)	319 (50.4%)	76 (36.0%)	63 (42.9%)	24 (27.0%)	
Pathological type						
LSC		51 (8.1%)	—	—	—	—
LAC		575 (90.8%)	—	—	—	
SCLC		7 (1.1%)	—	—	—	
TNM stages						
I		550 (86.9%)	—	—	—	—
II		32 (5.1%)	—	—	—	
III		30 (4.7%)	—	—	—	
IV		21 (3.3%)	—	—	—	

Abbreviations: LAC, lung adenocarcinoma; LSC, lung squamous cell carcinoma; SCLC, small cell lung cancer; TNM, tumor–node–metastasis.

### 2.2 Inclusion and exclusion criteria

Patients with pulmonary nodules were diagnosed as LC or benign lung disease by pathology examination. Exclusion criteria consisted of (1) patients with unclear LC staging and (2) patients with both adenocarcinoma cells and squamous cell carcinoma cells confirmed. In addition, the healthy examination group excluded patients, such as drug abuse, recent illness, recent hospitalization, recent surgery, pregnancy, and recent transfusion. We collected seven TAAbs and three tumor markers test results of healthy individuals who examined at the Ningbo Lihuili Hospital Physical Examination Center. The healthy samples were taken according to CLSI EP28‐A3c guideline.^19^

### 2.3 Sample collection and measurement

Five milliliters of blood was collected with a coagulation promoting tube (Zhejiang Gongdong Medical Device Co., Ltd.) and centrifuged at 1370 *g* for 10 min after collecting samples for 30 min. Three tumor markers (CYFRA21‐1, NSE, and SCCA) were detected within 12 h after separation. When not immediately tested, the serum was stored at −20°C for detection within 1 week. Seven TAAbs were detected within 8 h after separation. The serum was also stored at −20°C for detection within 1 week. Chemiluminescence immunoassay was used to detect the concentrations of the tumor markers, and ELISA was used to detect the concentrations of the TAAbs. A positive result was judged if the value of any one of seven TAAbs or three tumor markers was higher than the upper level of the reference interval. As a result, the combined detection was defined as positive. A negative result was judged if all of the values were lower than the upper level of the reference interval.

### 2.4 Instruments

Seven TAAbs were detected using assay kits (Hangzhou Cancer probe Biotech Company) and measured the levels of the TAAbs with Microplate Reader (ST360, Shanghai Kehua Biotechnology Co., Ltd.). The three tumor markers were detected using Unicel DxI800 (Beckman Coulter). The test was performed strictly according to the manufacturer's instructions. The positive criteria for each marker were as follows: SOX2 ≥ 10.3 U/ml, GAGE7 ≥ 14.4 U/ml, p53 ≥ 13.1 U/ml, PGP9.5 ≥ 11.1 U/ml, GBU4‐5 ≥ 7.0 U/ml, MAGE A1 ≥ 11.9 U/ml, CAGE ≥7.2 U/ml, CYFRA21‐1 ≥ 7.0 ng/ml, NSE ≥ 13 ng/ml, and SCCA ≥2.5 ng/ml.

### 2.5 Statistical analysis

The SPSS version 26.0 software was used for data analysis. The Kolmogorov–Smirnov test was used for non‐normal distribution analysis. Data were expressed as the median (interquartile range) (*M [P25, P75*]). The Kruskal–Wallis *H* test was used for comparisons of differences among groups, and the Mann–Whitney *U* test or Fisher's exact test was used for comparisons between two groups. The chi‐squared analysis was used to compare the positive rate. The ROC curve was applied to analyze the diagnostic efficiency. A *p* value <0.05 was considered statistically significant.

## 3 RESULTS

### 3.1 TAAb and tumor marker levels among four groups

Levels of p53, PGP9.5, SOX2, GAGE7, and CAGE in the LC group were higher than those of the other three groups. Additionally, the levels of p53, SOX2, and GAGE7 differed among the four groups (*p* < 0.05). CYFRA21‐1 and NSE expression levels also differed among the four groups (*p* < 0.05), while PGP9.5, GBU4‐5, MAGE A1, CAGE, and SCCA levels had no significant difference (*p* > 0.05) (Table 2).

**TABLE 2:** Levels of different markers among four groups [*M (P25, P75)*]

Markers	Lung cancer (*n* = 633)	Healthy examination (*n* = 211)	Benign lung disease (*n* = 147)	Other tumor (*n* = 89)	*H*	*p*
p53	2.4 (0.2, 1.7)	1.5 (0.1, 1.1)	1.9 (0.2, 1.9)	1.3 (0.2, 1.5)	9.745	0.021
PGP9.5	2.3 (0.1, 0.9)	1.7 (0.1, 0.5)	1.3 (0.1, 0.6)	1.1 (0.1, 0.5)	0.268	0.966
SOX2	2.6 (0.1, 1.8)	1.2 (0.1, 1.1)	2.0 (0.1, 2.1)	2.3 (0.1, 2.2)	8.492	0.037
GAGE7	4.3 (0.3, 2.7)	1.4 (0.3, 1.6)	1.9 (0.3, 2.5)	2.1 (0.3, 2.5)	9.180	0.027
GBU4‐5	2.2 (0.1, 2.0)	1.3 (0.1, 0.9)	2.3 (0.1, 2.4)	1.9 (0.1, 1.7)	16.721	0.001
MAGE A1	0.9 (0.0, 0.3)	0.2 (0.1, 0.1)	0.5 (0.0, 0.2)	1.7 (0.1, 0.5)	7.551	0.056
CAGE	1.8 (0.0, 0.5)	0.6 (0.1, 0.3)	1.2 (0.1, 0.5)	1.0 (0.1, 0.2)	0.628	0.890
CYFRA21‐1	3.2 (2.1, 3.9)	2.5 (1.9, 3.0)	2.6 (1.7, 3.5)	3.4 (1.7, 3.8)	25.268	0.000
NSE	3.2 (2.3, 3.7)	3.7 (2.9, 4.3)	3.1 (2.3, 3.6)	3.3 (2.6, 3.8)	53.561	0.000
SCCA	1.6 (0.9, 1.8)	1.5 (1.0, 1.8)	1.6 (0.9, 1.7)	1.8 (0.9, 2.0)	2.271	0.518

### 3.2 Positive rates of different markers among four groups

In the LC group, the positive rate of each marker was higher than that in other groups, and the positive rate of the combination was higher than that of a single test. The positive rate of the combined detection of the seven TAAbs was 29.1%. When combined with all markers, it rose to 37.8%, which was higher than that of benign lung disease (20.4%), healthy examination (16.6%), and other tumor (32.6%) groups. Compared with the control group (18.2%), the difference was statistically significant (*χ* ^2^ = 41.925, *p* < 0.001). In addition, the levels of GAGE7, CYFRA21‐1, and SCCA were statistically significant between the LC and benign lung disease groups, but the levels of the remaining markers were not statistically different. Notably, regardless of the combination of the TAAbs or TAAbs and tumor markers, the results were statistically significant and more meaningful than single detection (Table 3).

**TABLE 3:** Positive rates of different markers among four groups

Markers	Total (*n* = 1080)	Lung cancer (*n* = 633)	Healthy examination (*n* = 211)	Benign lung disease (*n* = 147)	Other tumor (*n* = 89)	*χ* ^2^	*p*1	*p2*	*p3*	*p4*
p53	35 (3.2)	27 (4.3)	4 (1.9)	3 (2.0)	1 (1.1)	4.338	0.219	0.206	0.113	0.237
PGP9.5	46 (4.3)	34 (5.4)	7 (3.3)	3 (2.0)	2 (2.2)	5.036	0.169	0.087	0.229	0.298
SOX2	48 (4.4)	37 (5.8)	2 (0.9)	5 (3.4)	4 (4.5)	9.376	0.025	0.237	0.003	0.606
GAGE7	35 (3.2)	32 (5.1)	0 (0.0)	0 (0.0)	3 (3.4)	22.720	0.000	0.005	0.000	0.791
GBU4‐5	92 (8.5)	65 (10.3)	9 (4.3)	12 (8.2)	6 (6.7)	7.770	0.051	0.441	0.008	0.295
MAGE A1	18 (1.7)	12 (1.9)	0 (0.0)	1 (0.7)	5 (5.6)	11.206	0.006	0.480	0.044	0.047
CAGE	45 (4.2)	35 (5.5)	2 (0.9)	5 (3.4)	3 (3.4)	8.775	0.032	0.292	0.005	0.610
7‐TAAbs	247 (22.9)	184 (29.1)	23 (10.9)	23 (15.6)	17 (19.1)	35.987	0.000	0.001	0.000	0.049
CYFRA21‐1	28 (2.6)	20 (3.2)	0 (0.0)	0 (0.0)	8 (9.0)	23.182	0.000	0.021	0.006	0.015
NSE	1 (0.1)	1 (0.2)	0 (0.0)	0 (0.0)	0 (0.0)	2.215	1.000	1.000	1.000	1.000
SCCA	104 (9.6)	68 (10.7)	16 (7.6)	7 (4.8)	13 (14.6)	8.649	0.033	0.027	0.184	0.279
SCCA+CYFRA21‐1 + NSE	127 (11.8)	84 (13.3)	16 (7.6)	7 (4.8)	20 (22.5)	21.719	0.000	0.004	0.027	0.021
7‐TAAbs+CYFRA21‐1	265 (24.5)	198 (31.3)	23 (10.9)	23 (15.6)	21 (23.6)	43.050	0.000	0.000	0.000	0.140
7‐TAAbs+SCCA	319 (29.5)	228 (36.0)	35 (16.6)	30 (20.4)	26 (29.2)	35.669	0.000	0.000	0.000	0.208
7‐TAAbs+NSE	247 (22.9)	184 (29.1)	23 (10.9)	23 (15.6)	17 (19.1)	35.987	0.000	0.001	0.000	0.049
7‐TAAbs+SCCA+NSE	319 (29.5)	228 (36.0)	35 (16.6)	30 (20.4)	26 (29.2)	35.669	0.000	0.000	0.000	0.208
7‐TAAbs+SCCA+CYFRA21‐1	333 (30.8)	239 (37.8)	35 (16.6)	30 (20.4)	29 (32.6)	41.925	0.000	0.000	0.000	0.344
7‐TAAbs+NSE + CYFRA21‐1	265 (24.5)	198 (31.3)	23 (10.9)	23 (15.6)	21 (23.6)	43.050	0.000	0.000	0.000	0.140
Combine all	333 (30.8)	239 (37.8)	35 (16.6)	30 (20.4)	29 (32.6)	41.925	0.000	0.000	0.000	0.344

*Note:* Values are expressed as No (%).

Abbreviations: 7‐TAAbs, seven autoantibodies; *p*1, four groups; *p*2, lung cancer versus benign lung disease; *p*3, lung cancer versus healthy examination; *p*4, lung cancer versus other tumor.

### 3.3 Diagnostic efficacy of different markers in lung cancer

**FIGURE 1:**
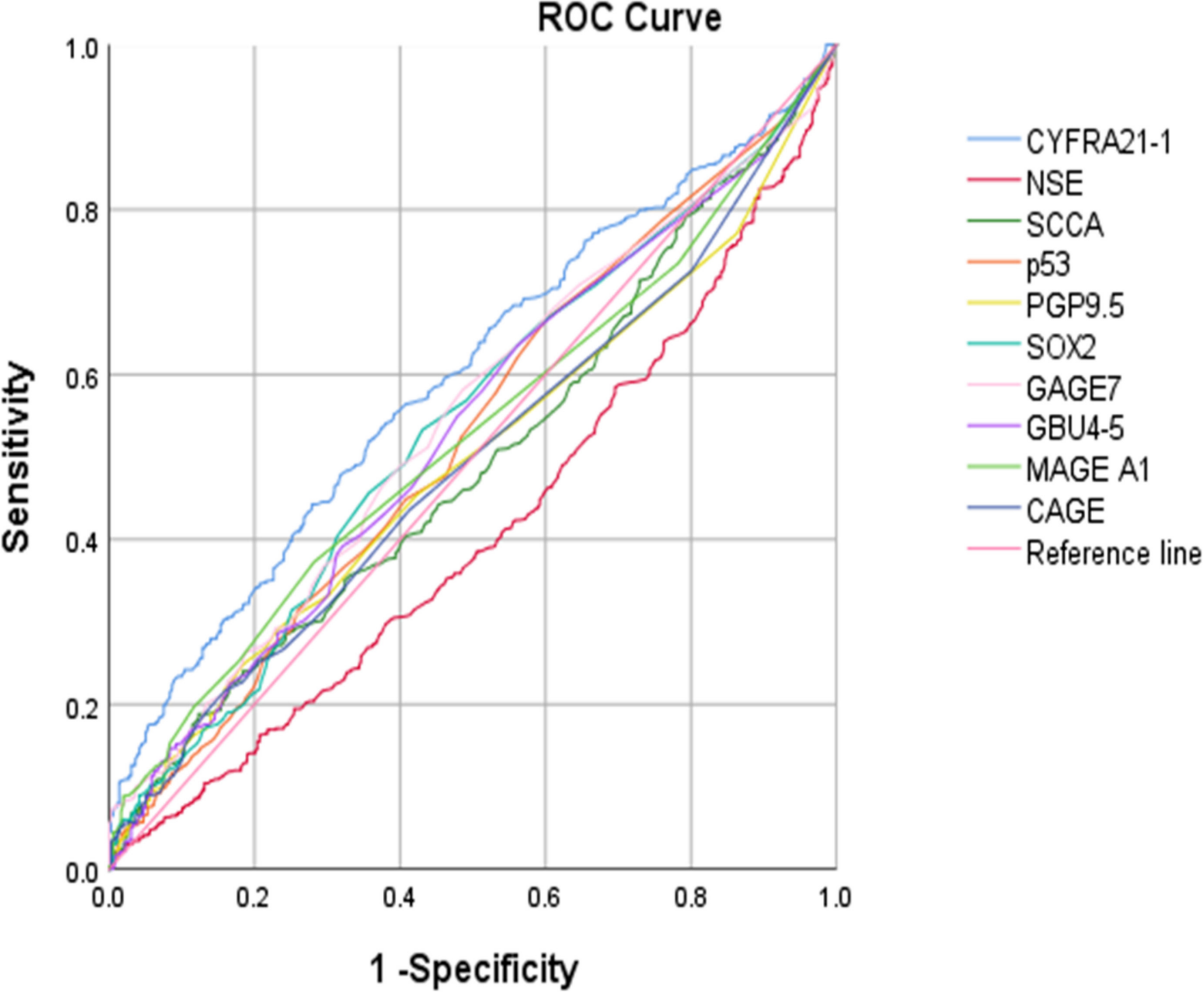
ROC curve of seven TAAbs and three tumor markers alone in lung cancer diagnosis

**FIGURE 2:**
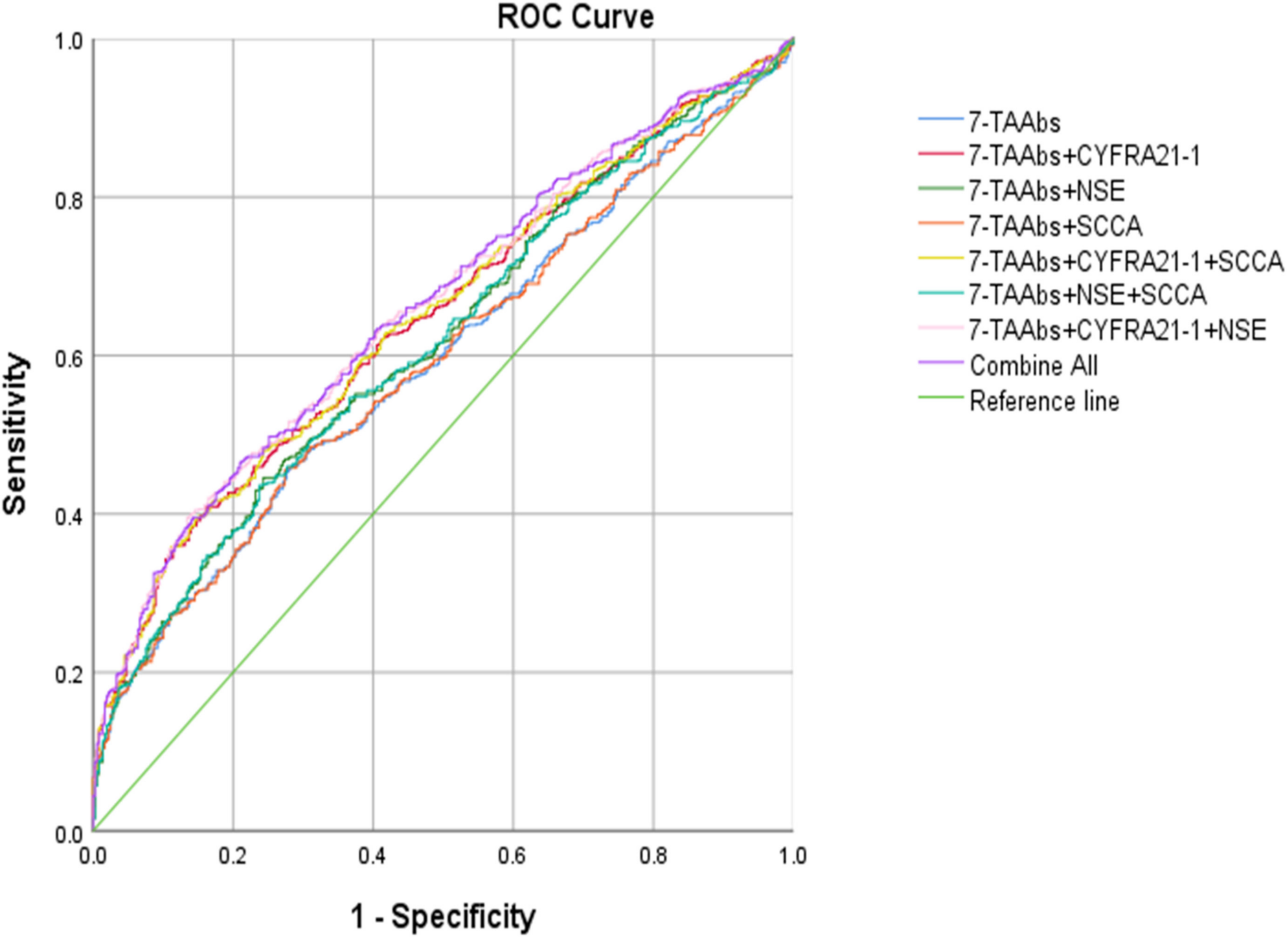
The ROC curve of the combined detection of seven TAAbs and three tumor markers in lung cancer diagnosis

Comparing the LC group with the control group, seven TAAbs and three tumor markers alone had high specificity but low sensitivity, and the combined detection could improve the sensitivity. The sensitivity of the combined detection of seven TAAbs was 29.07%, and the specificity was 87.15%. The detection sensitivity of the seven TAAbs combined with the three tumor markers (37.76%) was higher than that of the combined detection of seven TAAbs. The AUC of the combined detection of the seven TAAbs was 0.594, while the AUC of seven TAAbs combined with the three tumor markers was 0.660 (*p* < 0.05), and Youden's index was 0.196. Detection sensitivity of seven TAAbs combined with SCCA and CYFRA21‐1 was also 37.76%, and with AUC of 0.648 (Table 4, Figures 1 and 2).

**TABLE 4:** Diagnostic efficacy of different markers in lung cancer

Markers	PPV	NPV	Sensitivity	Specificity	AUC	Youden's index	*p*
p53	79.4%	36.7%	4.27%	98.04%	0.528	0.023	0.145
PGP9.5	81.0%	36.9%	5.37%	97.77%	0.495	0.031	0.786
SOX2	84.1%	37.1%	5.85%	98.04%	0.541	0.039	0.032
GAGE7	100.0%	37.3%	5.06%	100.00%	0.546	0.051	0.017
GBU4‐5	75.6%	37.2%	10.27%	94.13%	0.533	0.044	0.080
MAGE A1	92.3%	36.5%	1.90%	99.72%	0.526	0.016	0.176
CAGE	83.3%	37.0%	5.53%	98.04%	0.495	0.036	0.813
7‐TAAbs	80.0%	41.0%	29.07%	87.15%	0.594	0.162	0.000
CYFRA21‐1	100.0%	36.9%	3.16%	100.00%	0.597	0.032	0.000
NSE	100.0%	36.7%	0.16%	100.00%	0.411	0.002	0.000
SCCA	74.7%	37.2%	10.74%	93.58%	0.496	0.043	0.819
SCCA+CYFRA21‐1 + NSE	78.5%	37.9%	13.27%	93.58%	0.611	0.069	0.000
7‐TAAbs+CYFRA21‐1	81.1%	41.8%	31.28%	87.15%	0.646	0.184	0.000
7‐TAAbs+SCCA	77.8%	42.0%	36.02%	81.84%	0.594	0.179	0.000
7‐TAAbs+NSE	80.0%	41.0%	29.07%	87.15%	0.618	0.162	0.000
7‐TAAbs+SCCA+NSE	77.8%	42.0%	36.02%	81.84%	0.616	0.179	0.000
7‐TAAbs+SCCA+CYFRA21‐1	78.6%	42.6%	37.76%	81.84%	0.648	0.196	0.000
7‐TAAbs+NSE + CYFRA21‐1	81.1%	41.8%	31.28%	87.15%	0.659	0.184	0.000
Combine all	78.6%	42.6%	37.76%	81.84%	0.660	0.196	0.000

Abbreviations: 7‐TAAbs, seven autoantibodies; AUC, area under the curve; NPV, negative predictive value; PPV, positive predictive value.

### 3.4 Comparison of expression levels of each marker between LC and CG

**FIGURE 3:**
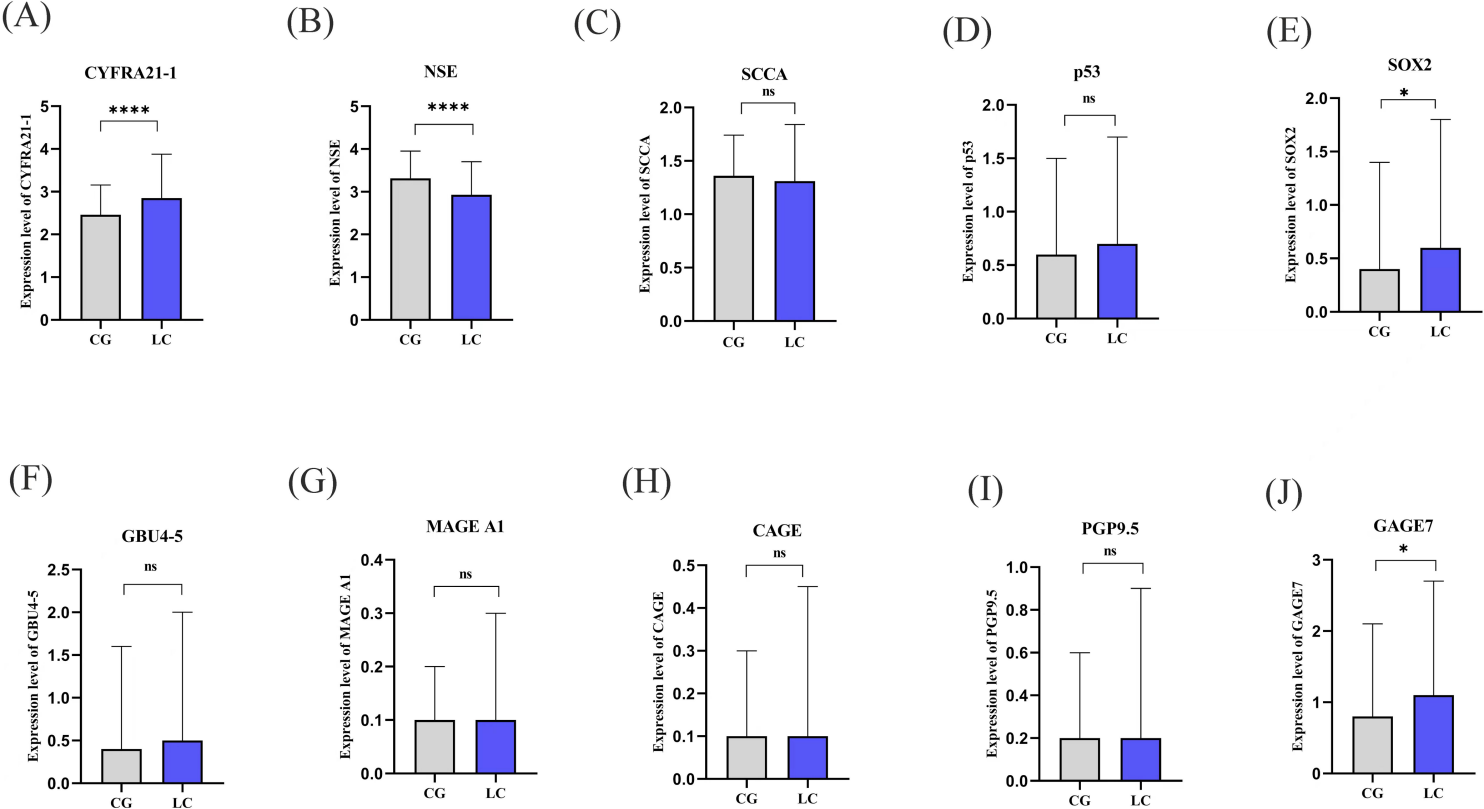
Comparison of expression levels of each marker between lung cancer group and control group. (A) Expression level comparison CYFRA21‐1 in LC and CG; (B) Expression level comparison NSE in LC and CG; (C) Expression level comparison SCCA in LC and CG; (D) Expression level comparison p53 in LC and CG; (E) Expression level comparison SOX2 in LC and CG; (F) Expression level comparison GBU4‐5 in LC and CG; (G) Expression level comparison MAGE A1 in LC and CG; (H) Expression level comparison CAGE in LC and CG; (I) Expression level comparison PGP 9.5 in LC and CG; (J) Expression level comparison GAGE7 in LC and CG; ns, *p* > 0.05; *p < 0.05; ****p < 0.001; CG, control group; LC, lung cancer

GAGE7, SOX2, CYFRA21‐1, and NSE displayed significant differences between the LC group and CG (*p* < 0.05), while p53, PGP9.5, GBU4‐5, MAGE A1, CAGE, and SCCA showed no significant difference between the two groups (*p* > 0.05) (Figure 3).

### 3.5 Comparison of positive rates among demographics of patients with LC

PGP9.5, p53, SOX2, GBU4‐5, GAGE7, CAGE, and NSE showed no significant differences in age or sex (*p* > 0.05), while MAGE A1 and SCCA displayed significant differences in sex (*p* < 0.05), but not in age (*p* > 0.05). Additionally, CYFRA21‐1 had a significant difference in age and sex (*p* < 0.05). Moreover, SOX2, MAGE A1, CYFRA21‐1, NSE, and SCCA were significantly different in pathological type and TNM (*p* < 0.05), while PGP9.5 and GAGE7 were significantly different in TNM (*p* < 0.05), but not in pathological type (*p* > 0.05). The remaining markers showed no significant differences in each characteristic (*p* > 0.05) (Table 5).

**TABLE 5:** Pathological characteristics in lung cancer

Parameters	Case (*n*)	p53	PGP9.5	SOX2	GAGE7	GBU4‐5	MAGE A1	CAGE	CYFRA21‐1	NSE	SCCA
Age (years)											
≥60	375	16 (4.3)	25 (6.7)	23 (6.1)	21 (5.6)	43 (11.5)	9 (2.4)	18 (4.8)	17 (4.5)	1 (0.3)	47 (12.5)
<60	258	11 (4.3)	9 (3.5)	14 (5.4)	11 (4.3)	22 (8.5)	3 (1.2)	17 (6.6)	3 (1.2)	0 (0.0)	21 (8.1)
*χ* ^2^		0.000	3.038	0.139	0.569	1.433	1.258	0.937	5.675	0.688	3.077
*p*		0.998	0.081	0.709	0.451	0.231	0.262	0.333	0.017	1.000	0.079
Gender											
Female	319	14 (4.4)	19 (6.0)	22 (6.9)	20 (6.3)	38 (11.9)	1 (0.3)	21 (6.6)	5 (1.6)	0 (0.0)	14 (35.7)
Male	314	13 (4.1)	15 (4.8)	15(4.8)	12 (3.8)	27 (8.6)	11 (3.5)	14 (4.5)	15 (4.8)	1 (0.3)	54 (17.2)
*χ* ^2^		0.024	0.433	1.292	1.976	1.886	8.657	1.367	5.328	1.016	27.076
*p*		0.877	0.511	0.256	0.160	0.170	0.003	0.242	0.021	0.496	0.000
Pathological type											
LAC	575	21 (3.7)	28 (4.9)	28 (4.9)	29 (5.0)	57 (9.9)	5 (0.9)	30 (5.2)	12 (2.1)	0 (0.0)	47 (8.2)
LSC	51	6 (11.8)	6 (11.8)	7 (13.7)	3 (5.9)	8 (15.7)	6 (11.8)	5 (9.8)	8 (15.7)	0 (0.0)	19 (37.3)
SCLC	7	0 (0.0)	0 (0.0)	2 (28.6)	0 (0.0)	0 (0.0)	1 (14.3)	0 (0.0)	0 (0.0)	1 (14.3)	2 (28.6)
*χ* ^2^		6.228	4.025	10.918	0.245	2.505	21.901	2.047	17.414	12.456	43.664
*p*		0.057	0.131	0.003	0.818	0.286	0.000	0.324	0.000	0.011	0.000
TNM stages											
I	550	21 (3.8)	23 (4.2)	27 (5.0)	22 (4.0)	58 (10.5)	6 (1.1)	27 (5.0)	8 (1.5)	0 (0.0)	50 (9.1)
II	32	3 (9.4)	7 (21.9)	4 (12.5)	3 (9.4)	5 (15.6)	3 (9.4)	4 (12.5)	5 (15.6)	0 (0.0)	8 (25.0)
III	30	2 (6.7)	0 (0.0)	1 (3.3)	5 (16.7)	1 (3.3)	1 (3.3)	3 (10.0)	4 (13.3)	0 (0.0)	6 (20.0)
IV	21	2 (9.5)	4 (19.0)	5 (23.8)	2 (9.5)	1 (4.8)	2 (9.5)	1 (4.8)	3 (14.3)	1 (4.8)	5 (23.8)
*χ* ^2^		5.095	19.757	11.995	10.656	2.814	14.276	4.907	29.021	10.884	13.590
*p*		0.118	0.000	0.005	0.009	0.393	0.002	0.122	0.000	0.033	0.002

*Note:* Values are expressed as No (%).

Abbreviations: LAC, lung adenocarcinoma; LSC, lung squamous cell carcinoma; SCLC, small cell lung cancer; TNM, tumor–node–metastasis.

## 4 DISCUSSION

Lung cancer is currently the most common tumor in the world, and a method of early diagnosis is crucial. For malignant tumors, early detection, diagnosis, and treatment are closely related to improving the survival rate of patients. In recent years, studies^20^, ^21^ on the efficacy of tumor markers and TAAbs alone or in combination in diagnosing LC have been increasing with varied results. For example, Wu et al. showed that the combination of CEA, CA153, and CYFRA21‐1 was more effective than single detection for the diagnosis of LC. They also found that CEA had the best AUC (0.665) for adenocarcinoma, and the AUC of CYFRA21‐1 (0.631) was better than CA153 and NSE.^22^ In addition, a study by Zang et al.^23^ showed that combined detection autoantibodies with tumor markers can improve the diagnosis of LC, and the AUC was 0.897. Furthermore, research by Huo et al.^24^ verified that the seven TAAbs played an important role in the early diagnosis of LC, and their sensitivity and specificity were 45.50% and 85.30%, respectively. However, research on whether tumor markers combined with autoantibodies can improve the early diagnosis of LC is limited.

Our study found that the efficacy of the single detection of seven TAAbs and three tumor markers was not ideal, while the diagnostic efficacy of their combination was significantly higher than that of each single detection. The sensitivity of the combined detection of seven TAAbs was 29.07%, and the AUC was 0.594. However, the sensitivity of the combined detection of seven TAAbs and three tumor markers was improved (37.76%). The results of a study by Ouyang et al. also showed that seven TAAbs combined with tumor markers could improve diagnostic efficiency. Specifically, they found that seven TAAbs combined with CEA and CYFRA21‐1 had better diagnostic performance than a single test, with sensitivity of 52.26% and specificity of 77.46%.^25^ Our results also showed that the combined detection performance of the three tumor markers was better than that of a single test. However, these results differed from those of Xu et al. who found that the combination of CYFRA21‐1, ProGRP, CEA, NSE, and SCCA did not improve the early diagnosis of LC in young patients. This could be because the patients in their experiment were younger, and tumor markers may differ for the diagnosis of young patients.^26^ In addition, we divided the lung cancer group into LAC and LSC. Compared with the CG, the sensitivity of seven TAAbs in the diagnosis of LSC was 47.06% and the AUC was 0.702. Meanwhile, that of LAC was 27.48% and the AUC was 0.584, which indicated that the diagnostic value of the seven TAAbs combined was higher in LSC than LAC. These findings were consistent with those of Huang et al.^27^ Therefore, we speculated that the seven TAAbs could play a vital role in the diagnosis of early‐stage LSC.

We also found that there were differences between the LC group and CG in the levels of SOX2, GAGE7, CYFRA21‐1, and NSE, which was consistent with the results of Huo et al.^24^ In this study, the positive rate in the LC group and CG was slightly different from the results of Chen,^28^ which could be due to our many cases of LAC.

Our results showed that PGP9.5, SOX2, GAGE7, GBU4‐5, MAGE A1, CYFRA21‐1, and SCCA differed among TNM stages, which was different from the results found by Wang et al.^16^ This could be because they selected more advanced stage specimens, while our experimental subjects were concentrated in the early stage. Additionally, their results showed that PGP9.5, SOX2, GBU4‐5, and CAGE were significant in LC. However, Luo et al. showed that p53 had better diagnostic efficacy in LC. Their results showed that there was no significant difference in the positive rate of each autoantibody in age or TNM stage (*p >* 0.05). However, the positive rate of MAGE A1 was statistically different among pathological types.^29^ This could be because MAGE A1 was closely related to the prognosis of LC, and studies have shown that CAGE is related to the metastasis of LC.

In summary, our experiment demonstrated that the combination of these seven TAAbs and three tumor markers is meaningful for the early diagnosis of LC, and the efficiency of combined detection is greater than that of single detection. However, our experiment has several limitations. First, the selected samples are limited, so there may be some selection bias. Second, the sample size of LSC or SCLC is small. Therefore, we will increase the tissue types of lung cancer for further research.

## 5 CONCLUSIONS

The combination of these seven TAAbs and three tumor markers could be useful in early diagnosis of LC, and the efficiency of combined detection was better than that of individual detection.

## AUTHOR CONTRIBUTIONS

MYY conceived and designed the study. LJ and XL collected and analyzed the clinical data. MYY and LJ were the major contributors in writing the manuscript. XFY and XGD reviewed and revised this manuscript.

## CONFLICT OF INTEREST

No potential conflict of interest was reported by the authors.

## Data Availability

The data analyzed during the current study are available from the corresponding author upon reasonable request.
